# Predator feeding choice on conspicuous and non-conspicuous carabid beetles: first results

**DOI:** 10.3897/zookeys.100.1525

**Published:** 2011-05-20

**Authors:** Teresa Bonacci, Pietro Brandmayr, Tullia Zetto Brandmayr

**Affiliations:** University of Calabria, Department of Ecology, I-87036 Rende (CS), Italy

**Keywords:** *Brachinus*, *Anchomenus*, anti-predatory strategies, warning signals, Coleoptera, Carabidae, laboratory tests

## Abstract

Insects use various types of behaviour, chemical defences, mimetic, aposematic or cryptic appearances as anti-predatory strategies. Among insects, carabid beetles of the genus *Brachinus* are distasteful prey because they discharge an irritating “cloud” of quinones when threatened. These beetles live in aggregations and adopt warning (conspicuous pattern) colours and chemicals to create a template that is easily learnt by predators. Another carabid beetle, *Anchomenus dorsalis*, mimics the colours and cuticular profile of *Brachinus* and is usually found in *Brachinus* aggregations. In this paper we report results from laboratory observations on feeding choice of the following natural predators - *Crocidura leucodon* (Insectivora: Soricidae), *Ocypus olens* (Coleoptera: Staphylinidae) and *Podarcis sicula* (Reptilia: Lacertidae) - on carabid beetle species. Comparing the number of attacks of predators towards aposematic and non-aposematic prey, there was a statistically significant preference towards non-aposematic prey.

## Introduction

Visual and chemical anti-predatory strategies influence trophic webs, as defensive substances (such as semiochemicals or ecomones) (sensu Pasteels 1977, 1982) play an important role (Pasteels et al. 1983) as deterrents against predators. A considerable amount of work has been done in evaluating anti-predatory strategies and in the identification of defence compounds in arthropods (Eisner 1970; Edmunds 1974; Guilford 1990; Alatalo and Mappes 1996; Gamberale and Tullberg 1998). Many animals use warning colours (or aposematism) to signal their unpalatability to potential predators (Cott 1940; Guilford 1990). In insects, aposematic colouration often co-occur with gregariousness (Edmunds 1974) increasing the effect of the aposematic signal (Poulton 1890; Cott 1940; Rowe and Guilford 1999; Riipi et al. 2001).

In Europe, *Anchomenus dorsalis* (Pontoppidan 1763), which produces methylsalicylate from its pygidial gland (Schildknecht 1970) as well as other chemicals (Bonacci et al., work in progress), is often found with species of the bombardier beetle genus *Brachinus* Weber, 1801(Wautier 1971; Juliano 1985; Zaballos 1985; Bonacci et al. 2004a; Mazzei et al. 2005; Zetto Brandmayr et al. 2006) and, like *Brachinus*, is brightly coloured (green-blue and red-brown). In terms of chemical defence, bombardier beetles are amongst the best protected insect taxa. When attacked, these beetles eject jets of fluid (with a loud popping sound) from a pair of gland openings on the tip of the abdomen, aiming their discharge with accuracy towards the threat. The active compounds of the secretion are 1,4-benzoquinones, p-benzoquinone and 2-methyl-p-benzoquinone, which are mixed explosively at the moment of ejection, and discharge at 100°C with an audible detonation (Schildknecht 1961; Aneshansley et al. 1969; Eisner 1970; Eisner and Aneshansley 1999; Eisner et al. 2005; Bonacci et al. 2008). A number of predators have been shown to be repelled by bombardier beetles, including ants, carabid beetles, praying mantids, spiders, frogs and toads (Eisner 1958, 2003; Eisner and Dean 1976; Thiele 1977; Dean 1980a, b; Bonacci et al. 2004a, b, 2006).

In this study we report results from laboratory observations on the number of attacks of natural insect predators: *Crocidura leucodon* (Hermann, 1780) (Insectivora: Soricidae), *Ocypus olens* (Müller**,** 1764), (Coleoptera: Staphylinidae) and *Podarcis sicula* Rafinesque, 1810 (Reptilia: Lacertidae) towards some species of carabid beetles.

## Material and methods

### The lizard Podarcis sicula

Eleven hand collected adult male lizards (*Podarcis sicula*) were used in this study (collected from Cosenza province, southern Italy). Lizards were kept in the laboratory under natural daylight conditions. They were maintained in plastic cages (55 cm length × 34 cm width × 33 cm height) with opaque sides. Prey used were four species of carabid beetles, two of which were conspicuous: *Brachinus sclopeta* (Fabricius, 1792)(N = 11), *Anchomenus dorsalis* (N = 11); and two non-conspicuous: *Amara anthobia* A. Villa & G. B. Villa, 1833(N = 11), *Amara aenea* (De Geer, 1774) (N = 11). The carabid beetles were collected by hand in the Crati Valley, Cosenza province, southern Italy.

Lizards were tested individually in an open arena (size: 28 cm length × 18 cm width × 16 cm height) with a lamp on a white plaster substrate. During the experiment temperature was maintained at 24–26°C. The trials were performed from June to July 2006. Each lizard was tested once by offering one individual of four prey species (*Brachinus sclopeta*, *Amara dorsalis*, *Amara aenea*, *Amara anthobia*) at the same time. Each carabid beetle was tested once. Before the beginning of the trial, each lizard was not fed for two days. The lizard to be tested was kept in the arena for 10 minutes before starting the trial. The trial began when the four prey individuals was put into the arena and lasted when the prey was ingested. If no predation occurred, the trial lasted for 30 minutes after the prey was put into the arena.

The behaviour of each lizard during the trial was recorded using a digital camcorder (Sony HDV 1080i). Attack delay and whether the carabid beetles were killed or refused were also recorded. Differences between the occurrences of attacking the different prey species were evaluated using the Chi-square test. Attack delay was evaluated using Mann-Whitney and Kruskal-Wallis tests, using the SPSS v.12.0 statistical package.

### The staphylinid beetle Ocypus olens

Ten adult male staphylinid beetles, *Ocypus olens*, were collected by hand in the field (Cosenza Province, Italy). Each beetle was kept in the laboratory in a climate chamber at 18–24°C under L/D: 18/6 photoperiod. Each individual was maintained in a plexiglas container (10×8×6 cm) with 2 cm of clayey soil. The trials were performed between September 2003 and July 2004. Each beetle was collected four days before the experiment and maintained until the end of the experiment.

The beetles were not fed the day before the trial. Each beetle was individually tested in the laboratory. During each trial, one staphylinid beetle was placed in an arena (10×8×6 cm), followed immediately by adding one of eight carabid prey species (see below). The observation period started immediately and lasted for 10 minutes (for a total of 80 minutes per staphylinid specimen) without a rest period between the interactions.

The order in which the different carabid beetle prey species were introduced to the arena was random. The trials were video-recorded with a Panasonic digital video-camera. We counted the number of attacks towards the different prey species. The model prey consisted of eight species of carabid beetles. Three species possess warning colours and chemical defences (*Brachinus sclopeta*, *Anchomenus dorsalis* and *Chlaenius velutinus* (Duftschmid, 1812)) and five are without these characteristics (*Steropus melas* (Creutzer, 1799), *Calathus fuscipes* (Goeze, 1777), *Pseudophonus rufipes* (De Geer, 1774)*, Poecilus cupreus* (Linné, 1758),and *Amara anthobia*). Attack frequency differences between species that possess warning colours and chemical defences, and those who do not possess these characteristics were evaluated using the Chi-square test in SPSS v.12.0.

### The shrew Crocidura leucodon

Two adult specimens of the shrew, *Crocidura leucodon* (1 male and 1 female), were collected by long worth traps (Pollino mountain, Calabria, 1200 m a.s.l.) in October 2002. The shrews were kept under laboratory conditions in plastic cages (55 cm length × 34 cm width × 33 cm height) with opaque sides under natural daylight conditions. Nine carabid species were used as prey; *Scybalicus oblongiusculus* (Dejeani, 1829), *Parophonus hispanus* (Rambur, 1838), *Steropus melas* and *Calathus montivagus* Dejeani, 1831 (without warning colours and chemical defences)and *Chlaenius chrysocephalus* (Rossi, 1790), *Anchomenus dorsalis*, *Brachinus brevicollis* (*= peregrinus*)(Apfelbeck 1904), *Brachinus sclopeta* and *Brachinus crepitans* (Linné, 1758)(withwarning colours and chemical defences). Shrew were tested individually in an open arena (size: 25 cm length × 15 cm width × 18 cm height) with plaster as a substrate and with low-light. Before the start of the trial, each shrew was starved for two days. The order in which the different carabid beetle prey species were introduced into the arena was random. The trials were video-recorded and the number of attacks towards the prey species was evaluated using the Chi-square test.

Carabid beetle nomenclature follows Vigna Taglianti (1993).

## Results

**Figure 1:**
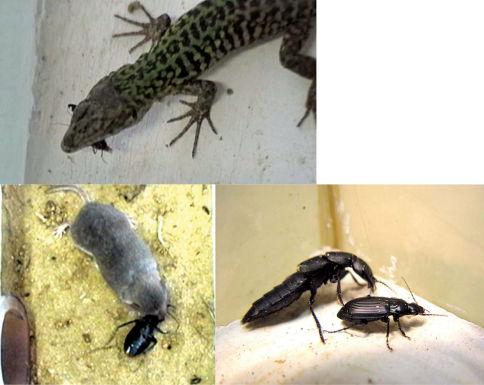
**a** Consumption of *Amara anthobia* by the lizard *Podarcis sicula* **b** attack on *Calathus fuscipes* by the staphylinid *Ocypus olens* **c** consumption of *Campalita maderae* by the shrew *Crocidura leucodon*.

We found a statistically significant preference towards non-conspicuous prey by the lizard *Podarcis sicula*. *Amara anthobia* and *Amara aenea* were attacked with high frequency (Fig. 1a), while *Brachinus sclopeta* and *Anchomenus dorsalis* with low frequency (*X2* = 23.76, DF = 3, P < 0.001). Non-conspicuous prey were captured and eaten without difficulty, but when *Brachinus sclopeta* or *Anchomenus dorsalis* were captured, lizards always tossed their heads and then rubbed their snouts on the soil. This is most likely because of the unpalatability of aposematic prey (Bonacci et al. 2008; Bonacci et al., work in progress).

The staphylinid beetle *Ocypus olens* reacted differently to chemically protected and unprotected carabids. Aposematic and chemically protected species (*Brachinus sclopeta*, *Anchomenus dorsalis* and *Chlaenius velutinus*) were attacked with lower frequency (*X2* = 23.56, DF = 1, P < 0.001) than species without these characteristics (*Poecilus cupreus*, *Pseudophonus rufipes*, *Calathus fuscipes*, *Steropus melas* and *Amara anthobia*). Larger carabid species (*Calathus velutinus* and *Steropus melas*) were attacked quicker than smaller-sized species (Fig. 1b) (Bonacci et al. 2006).

**Figure 2.**
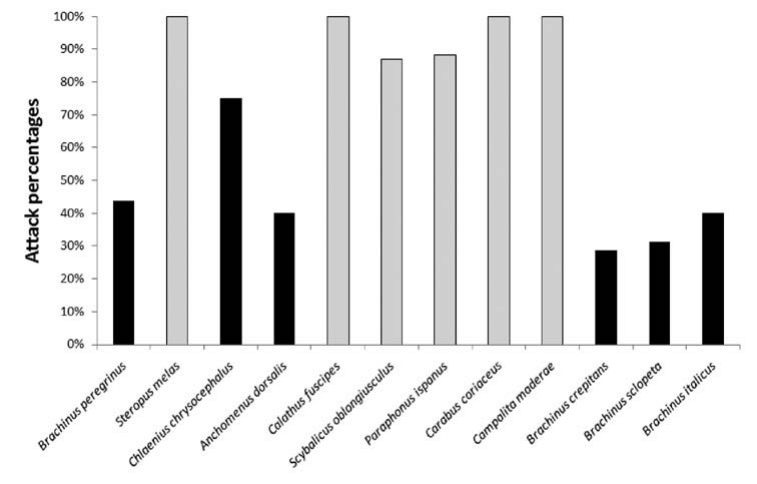
Percentage of attacks by *Crocidura leucodon* (Insectivora: Soricidae) on conspicuous and non-conspicuous carabid beetles. Black bars represent conspicuous species; grey bars represent non-conspicuous species.

The shrew *Crocidura leucodon* attacked and consumed all non-conspicuous and unprotected species of carabids, such as *Scybalicus oblongiusculus*, *Parophonus hispanus*, *Steropus melas* and *Calathus montivagus* (Fig. 1c). *Chlaenius chrysocephalus*, *Brachinus peregrinus*, *Brachinus crepitans*, *Brachinus sclopeta* and *Anchomenus dorsalis* were attacked infrequently (*X*2 = 35.25, DF = 1, P< 0.001) and with difficulty (Fig. 2) (Bonacci et al. 2004b).

## Discussion

**Figure 3.**
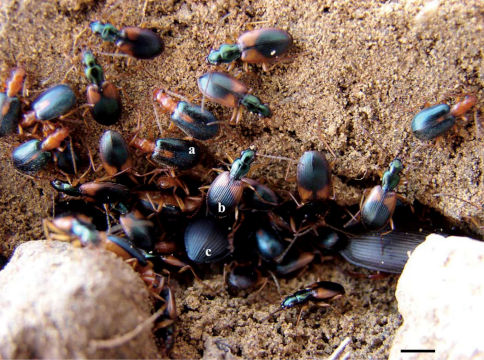
Interspecific aggregation of *Brachinus sclopeta* **a** *Anchomenus dorsalis* **b** and individuals of *Poecilus cupreus* **c**. Scale bar = 2 mm.

Our results support the hypothesis that conspicuous colouration and defence chemicals in gregarious carabid beetles can produce a sufficient aposematic signal to limit the attack by ambush and active predators. We found a statistically significant preference of predators for non-aposematic prey. Animals protected by chemical defence are often conspicuously coloured (Alcock 1979), since unpalatability is frequently coupled with warning signals (aposematic colours and odours) (Cott 1940; Tullberg et al. 2000). As such, edible prey may exploit the aversion of predators to warning-coloured species and evolve to resemble the model (Joron and Mallet 1998). Moreover, it is likely that unpalatability selects for gregariousness (Alatalo and Mappes 1996). Carabid beetles belonging to *Anchomenus dorsalis* use warning colouration and an odour pattern similar to that of *Brachinus sclopeta* (Bonacci et al. 2008; Bonacci et al. work in prep.) to trigger aversion in predators. In Müllerian mimicry, similarity does not necessarily need to be complete (Huheey 1988; Ihalainen et al. 2007), as in the case of *Anchomenus dorsalis* and *Brachinus sclopeta* (Fig. 3), which are quite similar in body size and colour pattern and live in conspicuous aggregations. These results suggest that colouration and chemicals (multimodal signals) used by the gregarious carabid beetles *Brachinus* spp. and *Anchomenus dorsalis* are an efficient anti-predatory strategy. In this case the quinones excreted by *Brachinus sclopeta* and other *Brachinus* speciesand the methylsalicilate (and probably other warning chemicals) produced by *Anchomenus dorsalis* can act as predator repellents. All predators tested here showed aversion towards *Brachinus* spp. and *Anchomenus dorsalis* individuals compared to non-conspicuous species (*Poecilus cupreus*, *Pseudophonus rufipes*, *Calathus fuscipes*, *Calathus montivagus*, *Steropus melas*, *Amara anthobia*, *Anchomenus aenea*, *Scybalicus oblongiusculus*, *Parophonus hispanus*).

As suggested by many authors, Müllerian mimicry may influence the diversity of defensive secretions of a species (Rettenmeyer 1970; Edmunds 1974; Pasteels et al. 1983) and in this case, *Anchomenus dorsalis* benefits from the different defence systems of *Brachinus* individuals. A similar anti-predatory system has been reported in several reviews concerning insect defence chemistry (Brower 1969; Blum 1981; Nishida 2002); also, Müllerian mimics are sympatric aposematic species that share the same or similar warning patterns (Wickler 1968). The anti-predatory strategies of *Brachinus* spp. and *Anchomenus dorsalis* appear to be supported by a combination of conspicuous colouration, defence chemicals and a gregarious habit.

Future chemical and behavioural work should attempt to determine whether species of conspicuous and chemical defense systems are recognizable by the constant emission of odours or by the emission of chemicals after contact with predators (Bonacci et al. work in progress).
